# Recruitment of Normal Stem Cells to an Oncogenic Phenotype by Noncontiguous Carcinogen-Transformed Epithelia Depends on the Transforming Carcinogen

**DOI:** 10.1289/ehp.1306714

**Published:** 2013-05-17

**Authors:** Yuanyuan Xu, Erik J. Tokar, Rachel J. Person, Ruben G. Orihuela, Ntube N.O. Ngalame, Michael P. Waalkes

**Affiliations:** National Toxicology Program Laboratory, Division of the National Toxicology Program, National Institute of Environmental Health Sciences, National Institutes of Health, Department of Health and Human Services, Research Triangle Park, North Carolina, USA

**Keywords:** cadmium, cancer stem cells, inflammatory factors, prostate, stem cells

## Abstract

Background: Cancer stem cells (CSCs) drive tumor initiation, progression, and metastasis. The microenvironment is critical to the fate of CSCs. We have found that a normal stem cell (NSC) line from human prostate (WPE-stem) is recruited into CSC-like cells by nearby, but noncontiguous, arsenic-transformed isogenic malignant epithelial cells (MECs).

Objective: It is unknown whether this recruitment of NSCs into CSCs by noncontact co-culture is specific to arsenic-transformed MECs. Thus, we used co-culture to examine the effects of neighboring noncontiguous cadmium-transformed MECs (Cd-MECs) and *N*-methyl-*N*-nitrosourea–transformed MECs (MNU-MECs) on NSCs.

Results: After 2 weeks of noncontact Cd-MEC co-culture, NSCs showed elevated metalloproteinase-9 (MMP-9) and MMP-2 secretion, increased invasiveness, increased colony formation, decreased *PTEN* expression, and formation of aggressive, highly branched duct-like structures from single cells in Matrigel, all characteristics typical of cancer cells. These oncogenic characteristics did not occur in NSCs co-cultured with MNU-MECs. The NSCs co-cultured with Cd-MECs retained self-renewal capacity, as evidenced by multiple passages (> 3) of structures formed in Matrigel. Cd-MEC–co-cultured NSCs also showed molecular (increased *VIM*, *SNAIL1,* and *TWIST1* expression; decreased *E-CAD* expression) and morphologic evidence of epithelial-to-mesenchymal transition typical for conversion to CSCs. Dysregulated expression of SC-renewal genes, including *ABCG2*, *OCT-4*, and *WNT-3*, also occurred in NSCs during oncogenic transformation induced by noncontact co-culture with Cd-MECs.

Conclusions: These data indicate that Cd-MECs can recruit nearby NSCs into a CSC-like phenotype, but MNU-MECs do not. Thus, the recruitment of NSCs into CSCs by nearby MECs is dependent on the carcinogen originally used to malignantly transform the MECs.

## Introduction

In recent years, the hypothesis of the existence of cancer stem cells (CSCs) has helped provide an explanation for tumor initiation, progression, therapeutic resistance, and tumor recurrence (Visvader and Lindeman 2008). CSCs have been found in or isolated from a variety of tumors (Visvader and Lindeman 2008). CSCs are typically a small subpopulation of the total tumor cells and, like normal stem cells (NSCs), have the capacity for self-renewal and an unlimited capacity for differentiation. CSCs share other characteristics with NSCs (Pardal et al. 2003): Like NSCs, CSCs reside in a niche (Borovski et al. 2011). Neighboring cells influence the differentiation and homeostasis of nearby stem cells (SCs) by releasing soluble factors, such as growth factors, chemokines, and cytokines, into the microenvironment (Abbott et al. 2008). However, compared with NSCs, CSCs have dysregulated self-renewal programming and are genetically unstable (Adams and Strasser 2008; Bomken et al. 2010; Pardal et al. 2003). The origin of CSCs is debated, but they may originate from adult SCs, partially differentiated progenitor cells, or even differentiated cells with multiple genetic and/or epigenetic alterations (Wang 2010). In addition, CSCs and more differentiated cancer cells may exist in a dynamic equilibrium, subject to a bidirectional conversion based on microenvironmental factors (Li and Laterra 2012).

SCs may play an important role in cancer induced by agents in the human environment. As exposure to the human carcinogen arsenic induces cancer *in vivo* or causes acquisition of a malignant phenotype *in vitro*, a survival selection of SCs occurs, causing an overabundance of CSCs (Sun et al. 2012; Tokar et al. 2010a, 2010b; Waalkes et al. 2008). In addition, arsenic directly transforms the NSCs into CSCs *in vitro* (Tokar et al. 2010a, 2013). Arsenic can also indirectly induce a CSC-like phenotype when NSCs are co-cultured with nearby, but noncontiguous, arsenic-transformed malignant epithelial cells (As-MECs), even though no actual physical cell-to-cell contact occurs (Xu et al. 2012). The noncontact conversion of NSCs to CSCs appears to involve soluble factors secreted by neighboring As-MECs and does not involve remnant arsenic (Xu et al. 2012). This recruitment of NSCs into CSCs by noncontact co-culture with MECs could have important general implications in tumor growth, invasion, and dissemination, but has only been observed with MECs originally transformed by arsenic (Xu et al. 2012).

Cadmium is a widespread environmental contaminant and an important human carcinogen [International Agency for Research on Cancer (IARC) 2012)]. Cadmium exposure is associated with increased risk of cancer, as well as cardiovascular, kidney, and bone disease [Agency for Toxic Substances and Disease Registry (ATSDR) 2008; IARC 2012]. In addition to occupational exposure, human exposure to cadmium mainly occurs through food consumption (in particular cereals and vegetables), tobacco use, and inhalation of ambient air (ATSDR 2008).

The mechanism of cadmium carcinogenesis likely differs from arsenic, another important human inorganic carcinogen (Achanzar et al. 2002; Tokar et al. 2010b). In transforming the same nontumorigenic human prostate epithelial cell line (RWPE-1), cadmium requires much less time than inorganic arsenic (8 vs. 29 weeks) (Achanzar et al. 2001, 2002). However, the production of CSC-like spheres, holoclones, and colony forming capacity induced by cadmium-transformation of RWPE-1 cells, which are all indicative of CSC formation, is much lower than that produced by arsenic-transformed RWPE-1 cells (Tokar et al. 2010b). Other evidence indicates that cadmium treatment leads to an overabundance of SCs and blocks differentiation during the malignant transformation of human breast epitheial cells (Benbrahim-Tallaa et al. 2009). Chronic cadmium exposure that induces cancer cell characteristics in human pancreatic cells also increases CSC-like cells (Qu et al. 2012). However, in one study, cadmium inhibited mouse prostate stem/progenitor cell proliferation and self-renewal (Jiang et al. 2011).

Although the recruitment of NSCs into CSCs by As-MECs could be an important general characteristic of MECs, to date, it has not been shown for malignant epithelia induced by carcinogens other than inorganic arsenic (Xu et al. 2012). Thus, in this study we examined whether cadmium-transformed MECs (Cd-MECs) had the ability to impact neighboring but noncontiguous NSCs. In addition, we also tested NSCs co-cultured with *N*-methyl-*N*-nitrosourea (MNU)-transformed MECs (MNU-MECs) to compare organic and inorganic carcinogens.

## Materials and Methods

*Cell lines and culture*. We used four cell lines. RWPE-1 cells are an immortalized nontumorigenic human prostate epithelia line derived from the nonneoplastic adult prostate (Bello et al. 1997). WPE-stem, a normal prostate stem cell (NSC) line isolated from RWPE-1 cells by single-cell dilution cloning, is well established as a NSC line showing multiple typical characteristics of urogenital system stem/progenitor cells (Tokar et al. 2005, 2010a). Cd-MECs (originally termed CTPE cells) were developed from RWPE-1 cells chronically exposed to cadmium (10 μM for 8 weeks). These Cd-MECs show loss of contact inhibition and elevated secretion of metalloproteinase-9 (MMP-9) and MMP-2 *in vitro* and form highly invasive tumors in mice (Achanzar et al. 2001). MNU-MECs (WPE1-NB26 cells), derived from second-generation tumors formed in nude mice by MNU-transformed RWPE-1 cells (10 μg/mL MNU for 1 hr for four cycles), are tumorigenic *in vivo* (Webber et al. 2001). Thus, all the cell lines used in the present study are isogenic.

Cells were maintained in low-calcium serum-free medium (keratinocyte serum-free medium; KSFM) containing 50 μg/mL bovine pituitary extract, 5 ng/mL epidermal growth factor, and 1% antibiotic–antimycotic mixture (Gibco, Rockville, MD). The effects of neighboring Cd-MECs or MNU-MECs on the NSCs were tested using co-culture transwell inserts, which separate the two types of cells by the width of approximately 50–100 normal prostate epithelia. These inserts do not allow cell-to-cell contact, but they do allow soluble factors to pass. Cell culture and co-culture were conducted as described previously (Xu et al. 2012). Control groups were NSCs co-cultured with untreated RWPE cells otherwise subjected to the same culture conditions.

*Free-floating sphere formation*. Floating sphere formation in culture is common for NSCs and CSCs (Ponti et al. 2005). After MEC co-culture, 1,000 SCs were plated in each well of an uncoated 6-well plate and fed every 48 hr. After 1 week, floating spheres and adherent cells were collected separately and stained with Trypan blue (Sigma-Aldrich, St. Louis, MO). Viable free-floating spheroids and adherent cells from each well were quantitated by microscopic visual counting and automated cell counter, respectively, and processed further as needed.

*MMP activity*. After MEC co-culture, SCs were grown alone; 48-hr conditioned medium was collected and cells were counted. We examined the activity of secreted MMP-9 and MMP-2 in medium by zymography (Tokar et al. 2005) and then adjusted to cell number. In some cases, we assessed MMP activity in conditioned medium from viable free-floating spheroid cells after MEC co-culture.

*Branched duct-like structures and serial passage*. After 2 weeks of co-culture, floating sphere cells were dissociated with 0.25% trypsin-EDTA, filtered with a 40-µm strainer to obtain the single cell suspension, resuspended in Matrigel (BD Biosciences, Bedford, MA) with KSFM (1:1, vol:vol), plated in the 24-well plate, and incubated at 37°C overnight to solidify before adding 1 mL KSFM. Medium was changed every 3 days. After 2 weeks, images were taken via inverted microscope. For serial passage, a single colony in Matrigel was dissociated into single cells and replated in Matrigel-KSFM mixture as described above.

*Anchorage-independent growth*. Colony formation in soft agar was performed as described (Tokar et al. 2005) on cells from free-floating spheres.

*Invasion assay*. Invasive capacity was assessed using a modified Boyden chamber assay as described by Bello et al. (1997).

*Real-time reverse transcription–polymerase chain reaction (RT-PCR)*. Total RNA, isolated from cultures with TRIzol (Invitrogen, Carlsbad, CA) and purified with RNeasy mini kit columns (Qiagen, Valencia, CA), was reverse transcribed to complementary DNA (cDNA) using Moloney murine leukemia virus reverse transcriptase (Applied Biosystems, Foster, CA). The resulting cDNAs were subjected to real-time RT-PCR for *PTEN* (phosphatase and tensin homolog), *VIM* (vimentin), *E-CAD* (E-cadherin), *SNAIL1* (SNAI1 snail family zinc finger 1), *TWIST1* (twist basic helix-loop-helix transcription factor 2), *ABCG-2* [ATP-binding cassette, sub-family G (WHITE), member 2], *OCT-4* (POU class 5 homeobox 1), and *WNT-3* (wingless-type MMTV integration site family, member 3) as described previously (Tokar et al. 2010a). Sequences of gene-specific primers (Sigma-Aldrich, St. Woodlands, TX) are available in Supplemental Material, Table S1 (http://dx.doi.org/10.1289/ehp.1306714).

*Western blots*. Western blots were conducted as described by Tokar et al. (2010b) using an antibody against PTEN (Abcam, Cambridge, MA). Protein bands were detected with the SuperSignal Chemiluminescence Substrate (Thermo Scientific, Rockford, IL).

*Immunofluorescence*. Immunofluorescence was conducted using Lab-Tek Chamber Slides (Electron Microscopy Sciences, Hatfield, PA) as described previously (Xu et al. 2012). After MEC co-culture, SCs were plated in chambers and fixed with acetone and methanol (1:1, vol:vol), blocked with normal horse serum [1:60 in phosphate-buffered saline (PBS) at room temperature for 1 hr), incubated with primary antibody [mouse anti-vimentin (Sigma-Aldrich, St. Louis, MO)] at 4°C overnight, and then incubated with secondary antibodies labeled with Alexa Fluor 568 (Molecular Probes, Eugene, OR) at room temperature for 1 hr. After washing with PBS, cells were stained with 4´,6´-diamideino-2-phenylindole (DAPI; Invitrogen, Eugene, OR) and examined via inverted fluorescence microscope.

*Transforming growth factor-*β*1 (TGF-*β*1) analysis*. After MEC co-culture for 2 weeks, medium was analyzed by enzyme-linked immunosorbent assay (ELISA) array using the Human Autoimmune Response Multi-Analyte ELISArray kit (Qiagen) and the Human TGF-β1 ELISA kit (R&D Systems, Minneapolis, MN). In a separate experiment, NSCs were treated with TGF-β1 (Peprotech, Rocky Hill, NJ) at 10 ng/mL for 96 hr and then tested for MMP secretion, cell morphology, and target gene expression.

*Statistical analysis*. Data represent the mean ± SE of at least three separate comparisons. For single comparisons with control, we used Student’s *t*-test. For multiple comparisons, we used the least significant differences (LSD) test after analysis of variance (ANOVA). A *p* < 0.05 was considered to be significant.

## Results

**Figure 1:**
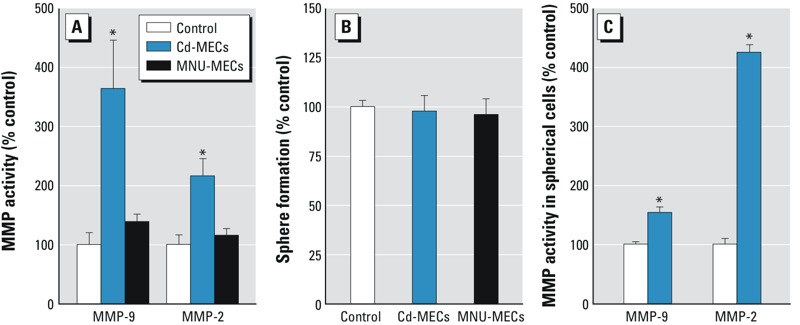
Secreted MMP activity and nonadherent sphere formation in SCs after 2 weeks of co‑culture with Cd‑MECs or MNU-MECs. (*A*) Secreted MMP activity in SCs. (*B*) Sphere formation. (*C*) Secreted MMP activity by spheroid cells. Data are presented as mean ± SE (*n* = 3). *p < 0.05, compared with control.

*Oncogenic transformation of NSCs*. In our previous work, we observed that noncontact co-culture with As-MECs could recruit NSCs into CSCs (Xu et al. 2012). To determine whether this was arsenic specific, we tested Cd-MECs and MNU-MECs in co-culture with NSCs. In previous studies of chemically induced malignant transformation (Achanzar et al. 2002; Tokar et al. 2010a, 2010b), cells identical to or isogenic with the NSCs used in the present study had increased activity of secreted MMP-9 200–450% that of the control and produced xenograph tumors in mice. In the present study, SCs in noncontact co-culture with Cd-MECs for 2 weeks showed increased secretion of both MMP-9 (360% of control) and MMP-2 (220% of control) (Figure 1A). Similar results were observed when the same NSCs were co-cultured with As-MECs (Xu et al. 2012). NSCs co-cultured with MNU-MECs showed no change in MMP secretion (Figure 1A).

Free-floating sphere formation is common for NSCs and CSCs, and we often see increased sphere formation in arsenic-induced CSCs. However, after noncontact co-culture with Cd-MEC or MNU-MEC, formation of floating spheres was unchanged (Figure 1B). However, floating spheres, often enriched in NSCs or CSCs, from Cd-MEC co-culture did show elevated MMP-9 secretion (150% of control) and MMP-2 (420% of control) secretion (Figure 1C), indicating an aggressive phenotype.

**Figure 2:**
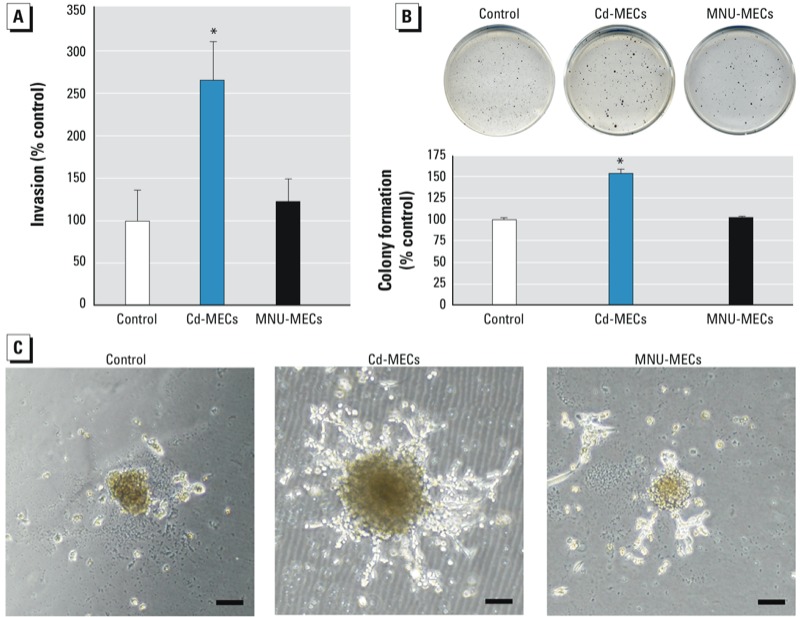
The cancer characteristics acquired by SCs after 2 weeks of co‑culture with Cd‑MECs or MNU-MECs. (*A*) Invasion capacity of spheroid SCs. (*B*) Colony formation of spheroid SCs. (*C*) Duct-like structures formed in Matrigel during 2 weeks by spheroid single SCs after co‑culture (bar = 200 μm). Quantitative data are presented as mean ± SE (*n* = 6). *p < 0.05, compared with control.

Cells derived from spheres generated from SCs co-cultured with Cd-MECs for 2 weeks showed a marked increase in invasion, indicating an aggressive phenotype, whereas cells co-cultured with MNU-MEC did not show enhanced invasion (Figure 2A).

Anchorage-independent growth (colony formation) is also common for SCs and indicates aggressive phenotype. After Cd-MEC co-culture, the spheroid-derived SCs formed more colonies than the control, but this did not occur after MNU-MEC co-culture (Figure 2B).

After Cd-MEC co-culture, sphere-derived SCs produced aggressive, highly branched, duct-like structures from single cells in Matrigel (Figure 2C). The structures formed after the Cd-MEC co-culture was serial passaged at least four times, confirming self-renewal capacity. Spheroid-derived SCs co-cultured with MNU-MEC or control SCs also formed structures in Matrigel that could be serial passaged, but the structures were small and showed minimal branching (Figure 2C). Thus, the SCs retained their self-renewal capacity after co-culture regardless of the carcinogen originally transforming the MECs, but they gained an aggressive phenotype only with Cd-MEC co-culture.

**Figure 3:**
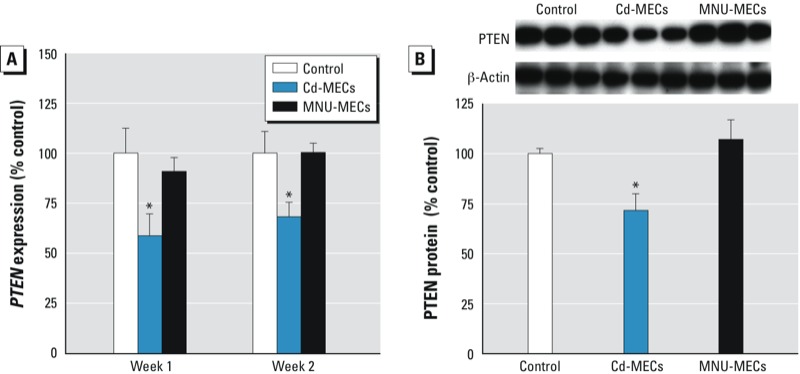
Expression of PTEN tumor suppressor gene. (*A*) Transcript levels of PTEN after 1 and 2 weeks of co‑culture. (*B*) Protein level after 2 weeks of co‑culture. Data are presented as mean ± SE (*n* = 3). *p < 0.05, compared with control.

*PTEN suppression and epithelial-to-mesenchymal transition*. PTEN is a tumor suppressor and has a key role in SC self-renewal and differentiation. A rapid suppression of *PTEN* expression occurred in SCs co-cultured with Cd-MECs (Figure 3), but not with MNU-MECs. Thus, at this point it was clear to us that Cd-MEC co-culture led to acquired cancer characteristics and that further in-depth study of Cd-MEC–co-cultured SCs was justified.

**Figure 4:**
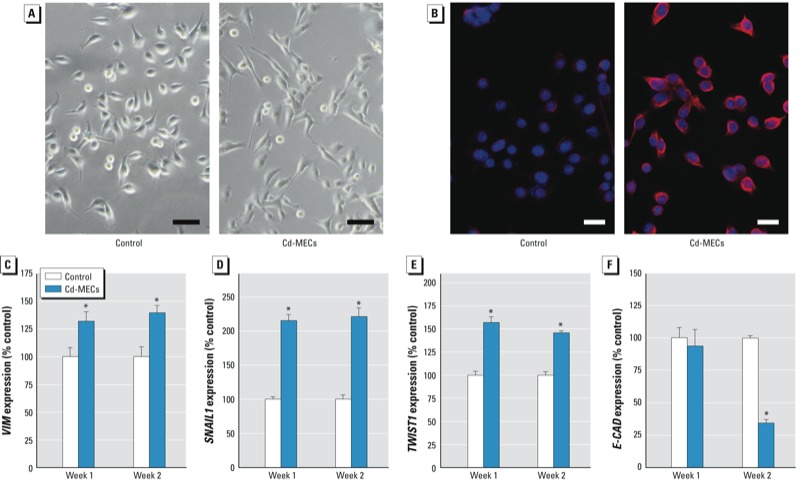
EMT (epithelial-to-mesenchymal transition) of SCs induced by 2 weeks of Cd‑MEC co‑culture. (*A*) SC morphology at week 2 (bar = 50 μm). (*B*) Protein levels of VIM mesenchymal cell marker shown by DAPI and VIM immunofluorescence (bar = 20 μm). (*C*) *VIM* transcript levels. (*D*) Transcript levels of the EMT inducer *SNAIL1*. (*E*) Transcript levels of the of EMT inducer *TWIST1*. (*F*) Transcript levels of the epithelial cell marker *E-CAD*. Quantitative data are presented as mean ± SE (*n* = 3). *p < 0.05, compared with control.

Epithelial-to-mesenchymal transition (EMT) is widely observed in cancer and plays a role in invasion and metastasis. After Cd-MEC co-culture, SCs took on a morphology (e.g., spindle shape) typical of mesenchymal cells, indicating that EMT had occurred (Figure 4A). With Cd-MEC co-culture, SCs showed a marked and widespread increase in VIM protein, a mesenchymal cell marker (Figure 4B), and increased *VIM* transcript (Figure 4C). Transcript of *SNAIL1* (Figure 4D) and *TWIST1* (Figure 4E), both EMT-inducing transcription factors, increased, whereas *E-CAD* transcript, an epithelial marker, decreased (Figure 4F).

**Figure 5:**
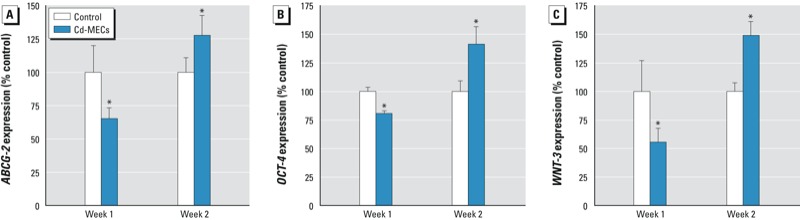
Expression of SC-related genes after 2 weeks of Cd‑MEC co‑culture shown by the deactivation and reactivation of (*A*) *ABCG‑2*, (*B*) *OCT‑4*, and (*C*) *WNT‑3*. Data are presented as mean ± SE (*n* = 3). *p < 0.05, compared with control.

*SC-related gene expression*. A “U-shaped” expression of the prostate SC-associated genes occurs in SCs during malignant transformation by either direct arsenic exposure or As-MEC co-culture (Tokar et al. 2010a; Xu et al. 2012). In the present study, we observed that Cd-MEC–co-cultured SCs also showed a U-shaped expression in the SC-related genes *ABCG-2*, *OCT-4*, and *WNT3*. The expression levels of these genes initially decreased with Cd-MEC co-culture at 1 week and rebounded above control after 2 weeks (Figure 5).

**Figure 6:**
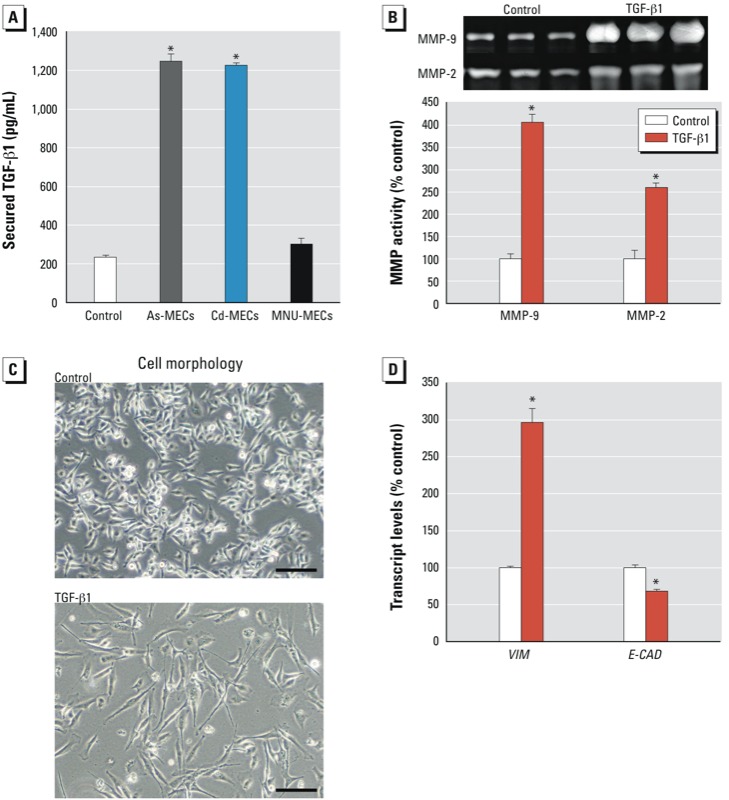
Possible involvement of TGF-β1 in recruitment of NSCs into CSCs by Cd-MEC or As‑MEC co‑culture. (*A*) Levels of secreted TGF-β1 in co‑culture medium at week 2. (*B–D*) Effects of direct treatment with TGF-β1 (10 ng/mL for 96 hr) on NSCs shown by (*B*) secreted MMP activity in TGF-β1–treated SCs, (*C*) SC morphology (bar = 100 μm), and (*D*) transcript levels of *VIM* and *E-CAD*. Quantitative data are presented as mean ± SE (*n* = 3). *p < 0.05, compared with control.

*Role of TGF-*β*1 in MEC co-culture*. We used ELISA to assess potential soluble factors that could be secreted by MECs and affect transformation by MEC co-culture. We observed high TGF-β1 protein levels in medium from As-MEC and Cd-MEC co-culture but not in medium from MNU-MEC co-culture (Figure 6A). Directly treating NSCs with TGF-β1 induced responses similar to those with Cd-MEC co-culture, such as increased MMP secretion (Figure 6B), EMT (Figure 6C), and gene expression changes such as decreased *E-CAD* and increased *VIM* transcript (Figure 6D).

## Discussion

CSCs have been hypothesized to be responsible for tumor initiation, progression, and metastasis (Visvader and Lindeman 2008). CSCs share many properties with NSCs, such as self-renewal capacity and differentiation potential, but in a distorted fashion. Clearly, the CSC microenvironment—which is composed of other cells, the extracellular matrix, and secreted factors—is key to their behavior (Moore and Lemischka 2006). Recently, we found that NSCs could be driven into a CSC-like phenotype by nearby, yet noncontiguous, isogenic As-MECs (Xu et al. 2012). In that study we used a noncontact co-culture technique that let only soluble, secreted factors from As-MECs interact with the NSCs over an approximate distance of 50–100 normal prostate cells (Xu et al. 2012). Essentially, this constitutes a recruitment of critical cells involved in tumor formation or replenishment by distant malignant epithelia, which was, in fact, made originally malignant by the important human carcinogen inorganic arsenic (Xu et al. 2012). In the present study, CSC recruitment also occurred with epithelial cells transformed by cadmium, another widespread human environmental carcinogen. For both arsenic and cadmium, this CSC recruitment could be an important mode of tumor expansion. Further, we tested Cd- and MNU-MECs, and found that only Cd-MEC co-culture could recruit NSCs into CSCs. The recruited cells kept basic SC characteristics, such as self-renewal ability and sphere-forming capacity, during the acquisition of the oncogenic phenotype. In contrast, MNU-MECs did not recruit NSCs into an oncogenic phenotype. This is further evidence that human prostate NSCs can be recruited into CSC-like cells via a microenvironment that contains substances secreted by MECs, but only by MECs originally transformed by some (inorganic arsenic and cadmium) but not all (MNU) carcinogens. This recruitment phenomenon may be a very important mechanism in extension and dissemination of chemically induced tumors, with cancer cells induced by some chemical carcinogens.

In the tumor microenvironment, surrounding tissues and tumors exchange information either directly through cell-to-cell contact or indirectly through chemical signaling from nearby cells (Bissell and Radisky 2001). In the co-culture system we used, there was no physical contact between SCs and MECs, making direct cell-to-cell information relay impossible. Although Cd-MECs were transformed by cadmium, which could accumulate and then possibly efflux, the cells were passed multiple times before the initial experiment; when tested, the amount of cadmium effluxed by Cd-MECs into the co-culture medium did not exceed background, as detected by atomic absorption spectrometry (data not shown). Thus, the recruitment of NSCs by Cd-MEC co-culture to CSC-like cells seems likely due to the secreted factors that pass from one chamber to the other. As-MECs (Xu et al. 2012) and Cd-MECs (present study) seem capable of inducing nearby NSCs into a cancer-like phenotype, whereas MNU-MECs are not. The reasons for these differences are not immediately apparent. MNU is a strong genotoxicant that acts by direct alkylation of DNA, resulting in mutations (Robbiano et al. 1989), and MNU induces malignant transformation *in vitro* in only a few hours (Webber et al. 2001). The mechanisms of arsenic and cadmium carcinogenesis are incompletely defined, although both likely involve multiple factors, including genetic and epigenetic changes, oxidative stress, and inflammation (IARC 2012). Chronic arsenic exposure induces stress and inflammation, which may contribute to cancer (Mo et al. 2011). Inflammation caused by chronic arsenic exposure can persist over time in spite of changes in exposure levels (Chen et al. 2007). Cadmium-induced tissue damage is often accompanied by inflammation involving cytokine production (Kundu et al. 2009). As-MECs and Cd-MECs were produced from chronic, low-level exposure (Achanzar et al. 2001, 2002) and likely involve chronic reactions that may possibly include production of inflammatory factors that could be secreted into the media, which in turn affect an SC microenvironment. Indeed, we previously saw that As-MECs secreted interleukin-6 (IL-6), which mimicked the CSC-recruiting effects of As-MEC co-culture (Xu et al. 2012). Inflammatory factors are important constituents of the tumor microenvironment, and inflammation is recognized as a key factor in cancer (Kim et al. 2009; Mantovani et al. 2008). Cd-MECs did not show elevated secretion of IL-6 (data not shown). However, in the present study, TGF-β1was highly secreted by both As-MECs and Cd-MECs but not by MNU-MECs, which did not recruit NSCs into CSCs. Studies have shown that TGF-β positively regulates tumor progression and metastasis (Bierie and Moses 2006). TGF-β can also induce EMT (de Graauw et al. 2010). Direct treatment of NSCs with TGF-β1 duplicated CSC recruitment by Cd-MECs in several ways—stimulating MMP secretion and EMT—thus suggesting that TGF-β1 may be another key signaling factor in the recruitment of NSCs into CSCs by some MECs, depending on the transforming carcinogen. The involvement of signaling factors in this CSC recruitment deserves further study, although there are very likely other important facets to this complex phenomenon.

EMT converts epithelial cells into cells with mesenchymal traits, involving loss of cell–cell adhesion, altered polarity, and increased motility (Kalluri and Weinberg 2009). EMT is important for tumor initiation, progression, and metastasis. Indeed, various epithelial cell lines, including prostate epithelial cells, undergo EMT during malignant transformation (Coppola et al. 2013). The EMT inducer TWIST1 promotes tumor development in mice and fosters malignant transformation of human mammary epithelial cells (Morel et al. 2012). Several studies have shown that EMT promotes the generation of CSCs (Mani et al. 2008; Morel et al. 2008). The induction of EMT by *SNAIL* and *TWIST* transfection or by TGFβ treatment in mammary epithelial cells results in cells with multiple CSC characteristics (Mani et al. 2008), consistent with our observation of *SNAIL* and *TWIST* overexpression during Cd-MEC co-culture recruitment of CSC-like cells. We also saw increases in the mesenchymal marker, *VIM*, and loss of the epithelial maker, *E-CAD*, indicating EMT. Thus, we found that NSCs acquired the morphology and molecular markers of EMT because they acquired a CSC-like phenotype from Cd-MEC co-culture. These data support the concept that EMT occurs during MEC recruitment of NSCs into CSCs.

During Cd-MEC co-culture, SCs showed decreased expression of the tumor suppressor gene *PTEN*. In prostate cancers, the loss of *PTEN* expression often occurs (Verhagen et al. 2006) and is related to the selection and expansion of SCs during malignant transformation (Tokar et al. 2010b). Similar to what occurred in As-MEC co-culture (Xu et al. 2012), some SC self-renewal genes, (*ABCG-2*, *OCT-4*, and *WNT-3*) showed dysregulated expression during the acquisition of CSC-like characteristics after Cd-MEC co-culture. *ABCG-2* is thought to be a survival factor of SCs, ultimately driving tumor growth (Dean et al. 2005). Both *OCT-4* and *WNT* regulate SC self-renewal, potentially drive the acquisition of CSC-like properties, and are linked to carcinogenesis (Hochedlinger et al. 2005; Vermeulen et al. 2010). This early loss and subsequent reactivation of critical SC-related gene expression occurred in arsenic-induced malignant transformation of WPE-stem cells (Tokar et al. 2010a) and transformation of hematopoietic SCs into leukemic SCs (Krivtsov et al. 2006). Dysregulated SC self-renewal programming is typical for oncogenesis (Pardal et al. 2003). Thus, the distorted expression of SC-related genes is likely an important feature of CSC formation from NSCs.

## Conclusions

Our data provide evidence that NSCs can acquire CSC-like characteristics via a microenvironment that contains soluble factors derived from MECs originally transformed by the human environmental carcinogen cadmium. Inflammatory factors such as TGF-β1 may play a key role in this recruitment. The recruitment of NSCs into CSCs by nearby MECs does not apply to all carcinogens that originally induce the MECs, and the reasons for this difference should be further explored. This CSC recruitment may be an important aspect of carcinogenesis that facilitates chemically induced tumor growth, invasion, and dissemination for some human carcinogens.

## Correction

In Supplemental Material, Table S1, originally published online, the GenBank accession numbers for *E-CAD* and *TWIST1* and the primers for *E-CAD* were incorrect. They have been corrected.

## Supplemental Material

(197 KB) PDF

## References

[r1] Abbott DE, Bailey CM, Postovit LM, Seftor EA, Margaryan N, Seftor RE (2008). The epigenetic influence of tumor and embryonic microenvironments: How different are they?. Cancer Microenviron.

[r2] Achanzar WE, Brambila EM, Diwan BA, Webber MM, Waalkes MP (2002). Inorganic arsenite-induced malignant transformation of human prostate epithelial cells.. J Natl Cancer Inst.

[r3] Achanzar WE, Diwan BA, Liu J, Quader ST, Webber MM, Waalkes MP (2001). Cadmium-induced malignant transformation of human prostate epithelial cells.. Cancer Res.

[r4] Adams JM, Strasser A (2008). Is tumor growth sustained by rare cancer stem cells or dominant clones?. Cancer Res.

[r5] ATSDR (Agency for Toxic Substances and Disease Registry). (2008). Toxicological Profile for Cadmium.. http://www.atsdr.cdc.gov/toxprofiles/tp.asp?id=48&tid=15.

[r6] Bello D, Webber MM, Kleinman HK, Wartinger DD, Rhim JS (1997). Androgen responsive adult human prostatic epithelial cell lines immortalized by human papillomavirus 18.. Carcinogenesis.

[r7] Benbrahim-Tallaa L, Tokar EJ, Diwan BA, Dill AL, Coppin JF, Waalkes MP (2009). Cadmium malignantly transforms normal human breast epithelial cells into a basal-like phenotype.. Environ Health Perspect.

[r8] Bierie B, Moses HL (2006). Tumour microenvironment: TGFβ: the molecular Jekyll and Hyde of cancer.. Nat Rev Cancer.

[r9] Bissell MJ, Radisky D (2001). Putting tumours in context.. Nat Rev Cancer.

[r10] Bomken S, Fiser K, Heidenreich O, Vormoor J (2010). Understanding the cancer stem cell.. Br J Cancer.

[r11] Borovski T, De Sousa EMF, Vermeulen L, Medema JP (2011). Cancer stem cell niche: the place to be.. Cancer Res.

[r12] Chen Y, Santella RM, Kibriya MG, Wang Q, Kappil M, Verret WJ (2007). Association between arsenic exposure from drinking water and plasma levels of soluble cell adhesion molecules.. Environ Health Perspect.

[r13] Coppola V, Musumeci M, Patrizii M, Cannistraci A, Addario A, Maugeri-Sacca M (2013). BTG2 loss and miR-21 upregulation contribute to prostate cell transformation by inducing luminal markers expression and epithelial-mesenchymal transition.. Oncogene.

[r14] Dean M, Fojo T, Bates S (2005). Tumour stem cells and drug resistance.. Nat Rev Cancer.

[r15] de Graauw M, van Miltenburg MH, Schmidt MK, Pont C, Lalai R, Kartopawiro J (2010). Annexin A1 regulates TGF-β signaling and promotes metastasis formation of basal-like breast cancer cells.. Proc Natl Acad Sci USA.

[r16] Hochedlinger K, Yamada Y, Beard C, Jaenisch R (2005). Ectopic expression of *Oct-4* blocks progenitor-cell differentiation and causes dysplasia in epithelial tissues.. Cell.

[r17] IARC (International Agency for Research on Cancer). (2012). Cadmium and cadmium compounds. IARC Monogr Eval Carcinog Risk Hum 100C:121–145.. http://monographs.iarc.fr/ENG/Monographs/vol100C/mono100C.pdf.

[r18] Jiang G, Xu L, Zhang B, Wu L (2011). Effects of cadmium on proliferation and self-renewal activity of prostate stem/progenitor cells.. Environ Toxicol Pharmacol.

[r19] Kalluri R, Weinberg RA (2009). The basics of epithelial-mesenchymal transition.. J Clin Invest.

[r20] Kim S, Takahashi H, Lin WW, Descargues P, Grivennikov S, Kim Y (2009). Carcinoma-produced factors activate myeloid cells through TLR2 to stimulate metastasis.. Nature.

[r21] Krivtsov AV, Twomey D, Feng Z, Stubbs MC, Wang Y, Faber J (2006). Transformation from committed progenitor to leukaemia stem cell initiated by MLL-AF9.. Nature.

[r22] Kundu S Sengupta S Chatterjee S Mitra S Bhattacharyya A 2009 Cadmium induces lung inflammation independent of lung cell proliferation: a molecular approach. J Inflamm (Lond) 6 19; 10.1186/1476-9255-6-19 [Online 12 June 2009] 19523218 PMC2702298

[r23] Li Y, Laterra J (2012). Cancer stem cells: distinct entities or dynamically regulated phenotypes?. Cancer Res.

[r24] Mani SA, Guo W, Liao MJ, Eaton EN, Ayyanan A, Zhou AY (2008). The epithelial-mesenchymal transition generates cells with properties of stem cells.. Cell.

[r25] Mantovani A, Allavena P, Sica A, Balkwill F (2008). Cancer-related inflammation.. Nature.

[r26] Mo J, Xia Y, Wade TJ, DeMarini DM, Davidson M, Mumford J (2011). Altered gene expression by low-dose arsenic exposure in humans and cultured cardiomyocytes: assessment by real-time PCR arrays.. Int J Environ Res Public Health.

[r27] Moore KA, Lemischka IR (2006). Stem cells and their niches.. Science.

[r28] Morel AP Hinkal GW Thomas C Fauvet F Courtois-Cox S Wierinckx A 2012 EMT inducers catalyze malignant transformation of mammary epithelial cells and drive tumorigenesis towards claudin-low tumors in transgenic mice. PLoS Genet 8 e1002723; 10.1371/journal.pgen.1002723 [Online 24 May 2012] 22654675 PMC3359981

[r29] Morel AP Lièvre M Thomas C Hinkal G Ansieau S Puisieux A 2008 Generation of breast cancer stem cells through epithelial-mesenchymal transition. PLoS One 3 e2888; 10.1371/journal.pone.0002888 [Online 6 August 2008] 18682804 PMC2492808

[r30] Pardal R, Clarke MF, Morrison SJ (2003). Applying the principles of stem-cell biology to cancer.. Nat Rev Cancer.

[r31] Ponti D, Costa A, Zaffaroni N, Pratesi G, Petrangolini G, Coradini D (2005). Isolation and *in vitro* propagation of tumorigenic breast cancer cells with stem/progenitor cell properties.. Cancer Res.

[r32] Qu W, Tokar EJ, Kim AJ, Bell MW, Waalkes MP (2012). Chronic cadmium exposure *in vitro* causes acquisition of multiple tumor cell characteristics in human pancreatic epithelial cells.. Environ Health Perspect.

[r33] Robbiano L, Parodi A, Venturelli S, Brambilla G (1989). Comparison of DNA alkylation, fragmentation, and repair in maternal and fetal tissues of pregnant rats treated with a single dose of ethyl methanesulfonate, ethyl-*N*-nitrosourea, *N*-nitrosodiethylamine, and methyl-*N*-nitrosourea.. Teratog Carcinog Mutagen.

[r34] Sun Y, Tokar EJ, Waalkes MP (2012). Overabundance of putative cancer stem cells in human skin keratinocyte cells malignantly transformed by arsenic.. Toxicol Sci.

[r35] Tokar EJ, Ancrile BB, Cunha GR, Webber MM (2005). Stem/progenitor and intermediate cell types and the origin of human prostate cancer.. Differentiation.

[r36] Tokar EJ, Diwan BA, Waalkes MP (2010a). Arsenic exposure transforms human epithelial stem/progenitor cells into a cancer stem-like phenotype.. Environ Health Perspect.

[r37] Tokar EJ, Person RJ, Sun Y, Perantoni AO, Waalkes MP (2013). Chronic exposure of renal stem cells to inorganic arsenic induces a cancer phenotype.. Chem Res Toxicol.

[r38] Tokar EJ, Qu W, Liu J, Liu W, Webber MM, Phang JM (2010b). Arsenic-specific stem cell selection during malignant transformation.. J Natl Cancer Inst.

[r39] Verhagen PC, van Duijn PW, Hermans KG, Looijenga LH, van Gurp RJ, Stoop H (2006). The *PTEN* gene in locally progressive prostate cancer is preferentially inactivated by bi-allelic gene deletion.. J Pathol.

[r40] Vermeulen L, De Sousa E, Melo F, van der Heijden M, Cameron K, de Jong JH, Borovski T (2010). Wnt activity defines colon cancer stem cells and is regulated by the microenvironment.. Nat Cell Biol.

[r41] Visvader JE, Lindeman GJ (2008). Cancer stem cells in solid tumours: accumulating evidence and unresolved questions.. Nat Rev Cancer.

[r42] Waalkes MP, Liu J, Germolec DR, Trempus CS, Cannon RE, Tokar EJ (2008). Arsenic exposure *in utero* exacerbates skin cancer response in adulthood with contemporaneous distortion of tumor stem cell dynamics.. Cancer Res.

[r43] Wang JC (2010). Good cells gone bad: the cellular origins of cancer.. Trends Mol Med.

[r44] Webber MM, Quader ST, Kleinman HK, Bello-DeOcampo D, Storto PD, Bice G (2001). Human cell lines as an in vitro/in vivo model for prostate carcinogenesis and progression.. Prostate.

[r45] Xu Y, Tokar EJ, Sun Y, Waalkes MP (2012). Arsenic-transformed malignant prostate epithelia can convert noncontiguous normal stem cells into an oncogenic phenotype.. Environ Health Perspect.

