# Supplementary material for: Recruitment of Normal Stem Cells to an Oncogenic Phenotype by Noncontiguous Carcinogen-Transformed Epithelia Depends on the Transforming Carcinogen
Source: Environ Health Perspect. 2013 May 17;121(8):944–50. doi: 10.1289/ehp.1306714 (PMC3734505; doi:10.1289/ehp.1306714)
Supplement: (197 KB) PDF [file ehp.1306714.s001.pdf]

Environmental Health Perspectives doi:10.1289/ehp.1306714

Supplemental Material

Recruitment of Normal Stem Cells to an Oncogenic Phenotype by Non-contiguous Carcinogen-transformed Epithelia Depends on the Transforming Carcinogen

Yuanyuan Xu, Erik J. Tokar, Rachel J. Person, Ruben Orihuela, Ntube N. Olive Ngalame, and Michael P. Waalkes

Table of Contents

Supplemental Material, Table S1. Genes and primers for real time RT-PCR………………page 2

Supplemental Material, Table S1. Genes and primers for real time RT-PCR

Gene	GenBank Accession No.	Primers (5'→ 3')
		Forward: TTCACATCCTACCCCTTTGCA
PTEN	NM_000314	Reverse: TCTGAGCATTCCCTCCATTCC
		Forward: CGCCAACTACATCGACAAGGT
VIMENTIN	NM_003380.3	Reverse: ACTTGCCTTGGCCCTTGAG
		Forward: GTAGCAGCTCCAGCGTCAAAT
E-CADHERIN	NM_000424	Reverse: TTGGAAGGCAGTGACTTGCA
		Forward: TGTCTGCGTGGGTTTTTGT
SNAIL1	NM_005985.3	Reverse: AGTCTGTCAGCCTTTGTCCTGTA
		Forward: GGATCAAACTGGCCTGCAA
TWIST1	NM_057179	Reverse: CCCCTCAGAGGAAGGATGAA
		Forward: CGGGTGACTCATCCCAACAT
ABCG2	NM_004827	Reverse: CTTAACCAAAGGCTCAGGATCTCA
		Forward: CCCCATTTCACCACACTCTACTC
OCT4	NM_002701	Reverse: CCAGAGCAGTGACAGGAACAGA
		Forward: GCCTGGTCCCCAAGCAA
WNT3	NM_030753	Reverse: GCTGGGCATGATCTCGATGT

All primers are from Sigma-Aldrich Corp., The Woodlands, TX.
